# Kainari, a Unique Greek Traditional Herbal Tea, from the Island of Lesvos: Chemical Analysis and Antioxidant and Antimicrobial Properties

**DOI:** 10.1155/2018/6802753

**Published:** 2018-02-26

**Authors:** Evangelia Bampali, Konstantia Graikou, Nektarios Aligiannis, Ioanna Chinou

**Affiliations:** Department of Pharmacognosy and Chemistry of Natural Products, Faculty of Pharmacy, School of Health Sciences, National and Kapodistrian University of Athens, Panepistimiopolis-Zografou, 15771 Athens, Greece

## Abstract

The chemical composition, as well as the total phenolic content (TPC) and the potential antioxidant and antimicrobial activity, of three Kainari-herbal tea samples from different areas of Lesvos Island (Greece) was evaluated. The rich aroma of the mixtures was studied through GC-MS, as well as through Headspace Solid-Phase Microextraction (HS-SPME)/GC-MS analyses. Cinnamon, clove, nutmeg, pepper, and ginger were identified as main ingredients, while, throughout the chemical analysis of the volatiles of one selected sample, several secondary metabolites have been isolated and identified on the basis of GC-MS as well as spectral evidence as eugenol, cinnamic aldehyde and myristicin, cinnamyl alcohol, alpha-terpinyl acetate, and *β*-caryophyllene. Furthermore, two food dyes, azorubine and amaranth, were also isolated and identified from the infusions. The total phenolic content was estimated and the free radical scavenging activity was determined by DPPH and ABTS assays and the antimicrobial activity of the extracts was tested showing a very interesting profile against all the assayed microorganisms. Due to its very pleasant aroma and taste properties as well as to its bioactivities, Kainari-herbal tea could be further proposed as functional beverage.

## 1. Introduction

For over thousand years, herbs and plants have played a major role in traditional and herbal based medicines. Herbal infusions and plants are the major source of phenolic compounds in our diet. It is known that phenolic compounds have many biological activities such as antioxidant and antimicrobial [1]. Due to the increasing popularity of a whole market for herbal dietary supplements and traditional medicines, we present in this study the chemical analysis and potential bioactivities of a unique herbal tea combination named “Kainari.”

Kainari is a combination (mixture) of several spices, in powder form, which is used as herbal tea, usually with an intense red color, traditionally prepared in the Greek island of Lesvos and used in folk medicine as a warming beverage in winter. Historically, it was brought to the island from Greek emigrants from Asia Minor in early 19th century. In Turkey, a similar herbal tea called “kaynar” (means in Turkish boiled) or Lohusa Serbeti (convalescence serbet) is used traditionally in childbirth to give energy to the new mothers and to boost their lactation [2].

The tradition of making “Lohusa Serbeti” after a birth probably is derived from Byzantine birth tradition (custom) where what was called “lochozema,” a nourishing broth, was given to new mothers [2, 3]. It is mentioned in ceremonial books that after the birth of an imperial child “Porphyrogenitus” and in one-week-long period of celebration, the preparation of “lochozema,” a nutritious “childbed soup like” broth, was offered in Constantinople's main streets. Then, as birth custom, it was given to all new mothers, to aid milk production and recovery [4, 5].

The exact history of “Lohusa Serbeti” is not known but the drink was certainly popular during the latter part of the Ottoman Empire (1299–1922), when the drink was given to new mothers, people that visited the new mother and with jugs of the drink also sent to the mother's friends, family, and cleric as a way of announcing the birth of her child. After drinking “Lohusa Serbeti” everybody present had to say a prayer asking God to bless the new mother with a plentiful supply of breast milk [2]. A fifteenth-century Turkish poem by Suleyman Celebi describes how Amine, mother of the prophet Muhammed, is given serbet to quench her thirst during labour [6].

It is also important the red color of the drink as it is a symbol in Turkey for good luck and joy [2], but also has a symbolic imperial importance [7]. Moreover, red color is considered apotropaic, by which is meant that red color is thought to ward off evil so by giving a red drink to a new lactating mother, protect both the mother and her baby [2].

To prepare “Lohusa Serbeti,” water, cinnamon sticks and cloves, and “lohusa” sugar (pink-red in color, natural red color, or red food dye) are boiled together, to be served hot in winter or cold during summer [2, 8, 9].

“Kainari,” as already referred to, arrived to Lesvos Island after 1922 and it is used as a pleasant, aromatic, tonic, warming beverage. The composition of the mixture differentiates from “Lohusa Serbeti” and in our days, Kainari infusion is prepared by adding hot water to half spoon of Kainari mixture, which is produced by mixing powders of several spices, while the exact composition of mixture is kept secret. Some of the ingredients that are usually part of the herbal tea combination are mainly cinnamon and clove and in smaller quantities other spices such as ginger, nutmeg, galangal, and pepper.

The objective of this research was to study, for the first time, to the best of our knowledge, the composition of three different Kainari samples from three villages of Lesvos Island (Greece), as well as to evaluate the total phenolic content (TPC) and the potential antioxidant and antimicrobial activity. Six volatile compounds and two azo food dyes have been isolated and identified. The total phenolic content was estimated by Folin-Ciocalteu method and the free radical scavenging activity was determined by DPPH and ABTS assays. Moreover, the antimicrobial activity of the samples was tested against six Gram-positive and Gram-negative bacteria, two oral pathogens, and three pathogenic fungi.

## 2. Materials and Methods

### 2.1. Materials

Three samples of Kainari were obtained from different areas of Lesvos Island: Mytilene (KN1), Paleochori (KN2), and Agiassos (KN3). Analytical grade solvents (pentane, methanol, and water) were used for the extractions and the chromatographic techniques. The chemicals used throughout this study such as 1,1-diphenyl-2-picrylhydrazyl (DPPH), 2,2-azino-bis-(3-ethylbenzothiazoline-6-sulfonic acid) (ABTS), Folin-Ciocalteu reagent, gallic acid, caffeic acid and Trolox powder, silica RP-18, and silica gel plates (Kieselgel 60 F254 20 × 20 cm) were purchased from Sigma-Aldrich and Merck.

### 2.2. Methods

#### 2.2.1. Preparation of Extracts

*(i) Preparation of Herbal Teas (KN1 Aq, KN2 Aq, and KN3 Aq)*. 0.4500 g of each of the Kainari samples was extracted using 200 mL of boiled distilled water left for 45 min at 25°C. The filtrates were freeze-dried (Alpha I5, Christ, Osterode am Harz, Germany) and stored in desiccator.

*(ii) Preparation of Methanol Extracts (KN1 Meth, KN2 Meth, and KN3 Meth). *0.9750 g of each of the Kainari samples was extracted using 400 mL of methanol for 2 hours at 25°C and after filtration was evaporated in a rotary evaporator (R-200, Büchi, Flawil, Switzerland) and stored at −20°C until further analysis.

*(iii) Preparation of Pentane Extracts. *0.4500 g of each of the Kainari samples was extracted using 200 mL of pentane for 2 hours at 25°C and after filtration was evaporated (R-200, Büchi, Flawil, Switzerland) and stored as previously.

#### 2.2.2. Isolation of Compounds

*(i) Isolation of Volatile Compounds*. The essential oil of sample KN2 was received by Clevenger distillation using 15.00 g of sample KN2. The distillate A (35 mL), consisting of essential oil and aromatic water, was collected in a flask, while 2 ml of pure essential oil B was collected separately and analyzed through GC-MS.

In the distillate A was added 2 g of NaCl and the solution was cooled in ice water and transferred to a separatory funnel with 50 mL of hexane. The aqueous layer A1 was discarded, and the organic layer A2 was evaporated via rotary evaporation (R-200, Büchi, Flawil, Switzerland) to yield an oily extract C. In the extract C 15 mL hexane and 25 mL of 5% NaOH were added for further liquid-liquid separation. The aqueous layer C1 contained the sodium salt of eugenol, while the eugenol acetate, *β*-caryophyllene, myristicin, and cinnamic aldehyde remained in the organic layer C2.

Layer C1 was then evaporated and cooled on ice, while 50 mL of 6 M HCl was added to the solution, ensuring that the solution was acidic and it was extracted with 15 mL hexane yielding eugenol, which was identified through NMR, GC-MS, and comparison with literature data [10].

The organic layer C2 was chromatographed through preparative thin layer chromatography on silica gel plates using cyclohexane/dichloromethane 30/70 v/v as mobile phase, yielding cinnamic aldehyde, myristicin, cinnamyl alcohol, alpha-terpinyl acetate, and *β*-caryophyllene. All compounds were identified through NMR and/or GC-MS and literature data [11, 12].

*(ii) Isolation of Azo Dyes. *The freeze-dried herbal teas of KN1 and KN2 (454 mg), which presented the same chromatographic profile, and the freeze-dried herbal tea of KN3 (450 mg) were separately further purified using a medium pressure liquid chromatography MPLC (Borosilikat 3.3 Code Number 28147, Büchi, Flawil, Switzerland) packed with reversed phase silica gel (RP18) and two Series 1 pumps with flow speed 8 mL/min, eluted with decreasing polarities: H_2_O/MeOH (100/0 to 0/100). Azorubine was isolated and identified from the water fraction of KN1-KN2 and amaranth from KN3. The isolated compounds were identified by NMR and literature data [13].

#### 2.2.3. Identification of Compounds

*(i) Headspace Solid-Phase Microextraction (HS-SPME)*. The rich aroma of the Kainari samples was studied through HS-SPME/GC-MS analyses [14]. The HS-SPME was performed with carboxen/polydimethylsiloxane coated fiber (75 *μ*m coating 57330-U, Supelco, Bellefonte, PA, USA) attached in a manual SPME fiber holder (Supelco). For SPME extraction, 10 mg of each sample in a glass vial (15 mL) closed with PTFE coated silicone rubber septum was used. The temperature in our experiment was set at 70–75°C and the vial with sample was placed on the hot-plate for 15 minutes. After that time, the fiber was exposed for 5 minutes and then it was transferred to perform GC-MS analysis.

*(ii) GC-MS Analysis. *The analyses were performed on a HP 6890 GC with 5973 MSD (Hewlett-Packard, Germany), with ionization energy 70 eV. The GC is equipped with a 1/10 split/splitless injector and a 30 m long DP5 capillary column, 0.25 mm internal diameter, and 0.25 mm thickness. The temperature in the injection sample was 200°C and gas was He and flow rate is 0.7 mL/min. Identification was made using the Wiley275 library and bibliographic data. The temperature programs that have been used are the following:

*Temperature Program I. *The initial temperature is 100°C and then rises at a rate of 4°C/min, up to a maximum temperature of 300°C. Total analysis time was 52 min. This program was used to analyze methanol and pentane extracts.

*Temperature Program II. *The initial column temperature is 50°C which is maintained for 3 minutes and then increased at a rate of 30°C/min to 150°C. From 150°C, it rises at a rate of 3°C/min up to 250°C, where it remains for 10 min. It was used for the HS-SPME analyses [10].

*Temperature Program III. *The initial column temperature is 60°C and then increases at a rate of 3°C/min to a maximum temperature of 280°C. Total analysis time was 93 min. It was used for the analysis of the essential oil B.

*(iii) Nuclear Magnetic Resonance (NMR).* ^1^H-NMR spectra were obtained on a Bruker DRX 400 instrument (400 MHz) using CD_3_OD, CDCl_3_, and DMSO-d6 as solvents and TMS as an internal standard.

#### 2.2.4. Antioxidant Properties

*(i) Total Phenolic Content (TPC)*. It was determined by using Folin-Ciocalteu method with slight modifications [15]. As referred to literature, 10 mg of each crude methanol extract or lyophilized herbal tea was dissolved in 1 mL of corresponding extracting solvent to produce the stock of sample solution. The lower concentrations of sample were prepared by diluting 100 *μ*L of the stock sample solution with 900 *μ*L methanol or distilled water. Then, 50 *μ*L of sample was put in a test tube and mixed with 60 *μ*L of Folin-Ciocalteu reagent, followed by an addition of 120 *μ*L of 35% sodium carbonate after 3 minutes. Then, the resulting mixture was incubated in dark at room temperature for an hour and absorbance was measured at 725 nm (UV-1700, Shimadzu Corporation, Kyoto, Japan) after incubation. Caffeic acid was used as calibrated standard and results were expressed as milligram Caffeic Acid Equivalent per gram of dry extract (mg CAE/g dry extract). The content of phenolics for each extract was determined in triplicate.

Alongside, 25 *μ*L of extracts or standard solution of gallic acid (2.5 to 100 *μ*g/mL) in DMSO was added to 125 *μ*L of a Folin-Ciocalteu solution (10% v/v), followed by 100 *μ*L of sodium carbonate (7.5% w/v) in a 96-well plate. The reagents were mixed and incubated for 30 minutes at room temperature protected from light and the absorbance was measured at 765 nm. Total phenolic content was expressed as mg Gallic Acid Equivalents per gram of dry extract (mg GAE/g dry extract). The content of phenolics for each extract was determined in triplicate.

*(ii) 1,1-Diphenyl-2-picrylhydrazyl (DPPH) Assay. *The DPPH radical scavenging assay was performed according to a previously described method [15]. The stock DPPH solution (314 *μ*M) was prepared in absolute ethanol and kept in dark at room temperature until its use. Gallic acid was used as a positive control (IC50: 4.5 *μ*g/mL) and the total extracts were diluted in DMSO at appropriate concentrations. Briefly, in a 96-well plate, 190 *μ*L of the DPPH solution and 10 *μ*L of gallic acid or samples were incubated for 30 min at room temperature protected from light and the absorbance was measured at 517 nm. A negative control containing 10 *μ*L DMSO and 190 *μ*L DPPH was performed each time, as well as blanks containing 10 *μ*L sample and 190 *μ*L EtOH.

*(iii) 2,2-Azino-bis-(3-ethylbenzothiazoline-6-sulfonic Acid) (ABTS) Assay. *The ABTS radical cation scavenging assay was performed using the method reported by Re et al. (1999) with slight modifications [16]. In brief, a stock ABTS aqua solution (7 mM) was reacted with potassium persulfate aqua solution (2.45 mM) and kept overnight in dark to yield a dark colored solution containing ABTS^•+^ radical cation. Trolox was used as a positive control (IC50: 8.3 *μ*g/mL) and the total extracts were diluted in DMSO at appropriate concentrations. Prior to use in the assay, the ABTS^•+^ radical cation was diluted with distilled water for an initial absorbance of about 0.700 (±0.02) at 734 nm. Afterwards, in a 96-well plate 100 *μ*l of ABTS^•+^ radical cation solution and 50 *μ*l of Trolox or samples were added, incubated for 10 min at room temperature, and protected from light and the absorbance was measured at 734 nm. A negative control containing 50 *μ*L DMSO and 100 *μ*L ABTS was performed each time, as well as blanks containing 50 *μ*L sample and 100 *μ*L dist. H_2_O.

The percentage of DPPH and ABTS scavenging was estimated by the following equation:

(1) $$\begin{array}{c} AA\%=\left\{\;\frac{\left[\left(A-B\right)-\left(C-D\right)\right]}{\left(A-B\right)}\;\right\}\times 100. \end{array}$$

*A* is the control (without sample), *B* is the blank (without sample, without DPPH/ABTS), *C*is the sample, and *D* is the blank sample (without DPPH/ABTS). IC50 values were estimated for the most active extracts.

For both experiments which referred to the free radical scavenging, all samples were analyzed in triplicate. Measurements were performed using a TECAN Infinite M200 PRO multimode reader (Tecan Group, Männedorf, Switzerland).

#### 2.2.5. Antimicrobial Bioassay

The methanol extracts and the herbal teas of Kainari were evaluated for their in vitro antimicrobial activities using the standard antibiotics netilmicin, amoxicillin, and clavulanic acid in order to evaluate the sensitivity of the tested bacteria, while 5-fluorocytosine and amphotericin were used as sensitivity controls of the tested fungi [17, 18]. A total of eleven microorganisms were assayed among which there were four Gram-positive bacteria,* Staphylococcus aureus* (ATCC 25923),* Staphylococcus epidermidis* (ATCC 12228),* Streptococcus mutans*, and* Streptococcus viridans*, and four Gram-negative bacteria,* Escherichia coli* (ATCC 25922),* Enterobacter cloacae* (ATCC 13047),* Klebsiella pneumoniae* (ATCC 13883), and* Pseudomonas aeruginosa* (ATCC 227853), as well as three pathogen fungi* Candida albicans* (ATCC 10231),* C. tropicalis* (ATCC 13801), and* C. glabrata* (ATCC 28838).

## 3. Results and Discussion

### 3.1. Results from HS-SPME/GC-MS Analyses

All three samples of “Kainari” from Mytilini, Paleochori, and Agiassos in Lesvos were studied for the first time to our knowledge. The rich aroma of the mixture was studied through Headspace Solid-Phase Microextraction (HS-SPME)/GC-MS analyses. Furthermore, the pentane and methanol extracts were also studied through GC-MS with respective results. The major constituents that have been determined through GC-MS analyses were cinnamic aldehyde, eugenol, myristicin, *β*-caryophyllene, curcumene, and zingiberene, which can be related to the possible contained spices as shown in Table 1.

Through analyses of all extracts of the three samples (herbal teas, methanol, and pentane extracts) and the simultaneous use of bibliography we conclude that the main components in all three samples are cinnamon (~6/10) and clove (~3/10) and the remaining part (1/10) corresponds to both nutmeg and ginger.

It is remarkable that, according to the analyses of the volatiles in KN3, they were not identified as the main components of ginger* (Zingiber officinale)* and consequently we assume that ginger is not included in the mixture or it is included in small quantities. As for the presence of cardamom and pepper, due to the lack of some of their main metabolites (some of them exist in other contained plants at the same time), we conclude that if they are present they will be in small quantities.

### 3.2. Isolation and Determination of Compounds

Six compounds were isolated from the essential oil of sample *Κ*N2 by applying a series of liquid-liquid extractions based on acid-base chemical reactions, as well as with preparative TLC: eugenol, cinnamic aldehyde, and myristicin were determined by NMR spectroscopy and GC-MS analysis, while cinnamyl alcohol, alpha-terpinyl acetate, and *β*-caryophyllene were determined only by GC-MS analysis.

Also, two food dyes were isolated and identified as parts of the family of azo colors. Samples KN1 and KN2 contained the same food color azorubine [19], while sample KN3 contained amaranth [11].

The azo colors are compounds bearing the functional group R-N = N-R′, where R and R′ may be either aryl or alkyl. Azo dyes are one of the most important chemical categories of dyes which are used for the coloring of natural and synthetic fibers, candies, and cosmetics but also food and beverages [20, 21]. Many azo dyes are nontoxic, although some of them have been accused for allergic reactions and increasing hyperactivity in children [22].

The two isolated compounds are being referred to EFSA as amaranth (E123) and azorubine or carmoisine (Ε122) and are well-known food dyes [19, 23].

### 3.3. Antioxidant Properties

The total phenolic content (TPC) of the three samples was notably high, probably due to the known rich phenolic concentration of all spices that they contain [24, 25]. The sample KN3 revealed the higher concentration of phenolic content (Table 2).

The free radical scavenging activities of herbal teas and methanol extracts were determined by DPPH and ABTS assays (Table 3). All extracts showed significant antioxidant activity performing 30–64% inhibition at DPPH assay (50 *μ*g/mL), as well as 26–48% inhibition at ABTS assay (10 *μ*g/mL). The herbal tea KN3 performed the higher inhibition at DPPH (64.7%) and at ABTS (46.9%) which is in accordance with the high TPC. Also, the methanol extracts of KN1 and KN3 showed high inhibition.

### 3.4. Antimicrobial Bioassay

According to in vitro antimicrobial tests (Table 4) against two Gram-positive bacteria (*S. aureus* and* S. epidermidis*) and four Gram-negative ones (*E. coli*,* E. cloacae, K. pneumoniae, *and* P. aeruginosa*), as well as against oral pathogens* (S. mutans and S. viridans)* and three human pathogenic fungi (*Candida albicans, C. tropicalis, *and* C. glabrata*), the herbal teas exhibited a broad spectrum of strong antimicrobial activity, while KN3 herbal tea appeared to be the most active against all tested microorganisms, which can be explained by the high percentage of cinnamic aldehyde and *β*-caryophyllene that appears in herbal tea of KN3 as shown in Table 1.

## 4. Conclusion

In conclusion, the chemical composition, as well as the total phenolic content (TPC) and the potential antioxidant and antimicrobial activity, of three Kainari-herbal teas from different areas of Lesvos Island (Greece) was evaluated. All samples exerted very broad and strong antimicrobial activities against almost all Gram-positive and Gram-negative human pathogenic bacteria, as well as strong antioxidant activities.

According to the determined chemical analysis and total phenolic content, it could be concluded that “Kainari” herbal infusion, except its very pleasant taste, could be also further used as a functional herbal beverage, due to its beneficial properties, strong antioxidant, and antimicrobial activities for consumers. Certainly, more studies along the potential further bioactivities, as well as the entire process of the production of stable batches of “Kainari” combination at industrial level, have to be designed in future.

## Figures and Tables

**Table 1:** Comparative table for the powders of KN1, KN2, and KN3 with HS-SPME.

Compound	% area KN1	% area KN2	% area KN3	Possible contained plant
Cinnamic aldehyde	9.88	10.00	15.19	*Cinnamomum cassia*
Safrole	<1%	<1%	-	*Myristica fragrans, Piper nigrum*
Camphene	<1%	2.78	-	*Myristica fragrans, Piper nigrum*
Eugenol	34.50	30.18	22.71	*Syzygium aromaticum, Cinnamomum cassia, Myristica fragrans, Piper nigrum*
*α*-Copaene	1.88	4.16	10.81	*Syzygium aromaticum, Piper nigrum*
*β*-Caryophyllene	15.61	30.18	33.16	*Cinnamomum cassia, Syzygium aromaticum, Piper nigrum Myristica fragrans, Elettaria cardamomum*
*α*-Humulene	2.50	3.77	3.59	*Syzygium aromaticum, Piper nigrum, Elettaria cardamomum*
Ar-Curcumene	1.94	1.66	-	*Zingiber officinale*
Zingiberene	1.13	1.26	-	*Zingiber officinale*
Myristicin	3.34	-	-	*Myristica fragrans*

**Table 2:** Total phenolic content of the herbal teas of KN1, KN2, and KN3.

	mg CAE/gr dry extract	mg GAE/gr dry extract
Sample KN1 aq	308.29	115.2
Sample KN2 aq	320.31	149.2
Sample KN3 aq	383.41	184.5

**Table 3:** % inhibition at DPPH and ABTS assay of herbal teas and methanol extracts.

	DPPH	ABTS
	% inhibition 50 *μ*g/mL	% inhibition 10 *μ*g/mL
KN1 aq	37.4 ± 0.1	28.4 ± 0.1
KN2 aq	50.2 ± 1.4	38.8 ± 1.3
KN3 aq	64.7 ± 0.3	46.9 ± 0.9
KN1 meth	64.6 ± 0.3	48.2 ± 0.7
KN2 meth	40.9 ± 0.7	26.9 ± 0.7
KN3 meth	61.4 ± 0.1	40.3 ± 1.4

**Table 4:** Antimicrobial activity of herbal teas and methanol extracts.

	Samples	*S. aureus*	*S. epidermidis*	*P. aeruginosa*	*E. cloacae*	*K. pneumoniae*	*E. coli*	*S. mutans*	*S. viridans*	*C. albicans*	*C. tropicalis*	*C. glabrata*
Kainari	KN1 aq	16/0.09	17/0.04	14/0.13	14/0.15	13/0.10	15/0.17	13/0.30	14/0.39	12/0.40	13/0.39	13/0.40
	KN1 meth	11/0.70	11/0.73	10/0.90	10/0.93	10/0.89	10/0.95	nt	nt	11/0.42	13/0.33	12/0.31
	KN2 aq	17/0.04	17/0.05	15/0.10	15/0.13	15/0.09	15/0.12	15/0.15	16/0.16	12/0.43	13/0.40	13/0.42
	KN2 meth	10/0.72	11/0.68	10/0.86	10/0.87	10/0.92	09/0.98	nt	nt	10/0.53	11/0.44	12/0.42
	KN3 aq	**18/0.02**	**19/0.01**	**16/0.06**	**16/0.05**	**16/0.05**	**15/0.08**	**17/0.07**	**18/0.03**	**13/0.39**	**13/0.31**	**13/0.29**
	KN3 meth	10/0.72	11/0.68	10/0.86	10/0.87	10/0.92	09/0.98	nt	nt	12/0.38	13/0.33	13/0.34

	5-Fluorocytosine	nt	nt	nt	nt	nt	nt	nt	nt	0.1 · 10^−3^	1 · 10^−3^	10 · 10^−3^
	Amphotericin	nt	nt	nt	nt	nt	nt	nt	nt	1 · 10^−3^	0.5 · 10^−3^	0.4 · 10^−3^
	Amoxicillin	2 · 10^−3^	2 · 10^−3^	2.4 · 10^−3^	2.2 · 10^−3^	2.8 · 10^−3^	2 · 10^−3^	nt	nt	nt	nt	nt
	Netilmicin	4 · 10^−3^	4 · 10^−3^	8.8 · 10^−3^	8 · 10^−3^	8 · 10^−3^	10 · 10^−3^	nt	nt	nt	nt	nt
	Clavulanic acid	2 · 10^−3^	2 · 10^−3^	2.4 · 10^−3^	2.2 · 10^−3^	2.8 · 10^−3^	2 · 10^−3^	nt	nt	nt	nt	nt
